# Lessons Learned in the Early Adoption of FaME Across Ireland: A Convergent Mixed‐Methods Implementation Study (FaME Ireland)

**DOI:** 10.1111/hex.70860

**Published:** 2026-09-07

**Authors:** Scott Rooney, Leanne Ahern, Edel Brennan, Eibhlis Cahalane, Vanda Cummins, Caroline Eldridge, Frances Horgan, Dawn A. Skelton, Éidín Ní Shé, Katherine Thackeray, Boglarka Vig, Ruth McCullagh

**Affiliations:** ^1^ Research Centre for Health (ReaCH) Glasgow Caledonian University Glasgow UK; ^2^ Discipline of Physiotherapy, School of Clinical Therapies University College Cork Ireland; ^3^ Primary Care Services, Health Services Executive West and Northwest Ireland; ^4^ Primary Care Services Health Services Executive South West Ireland; ^5^ Primary Care Services Health Services Executive Dublin and North East Ireland; ^6^ School of Physiotherapy Royal College of Surgeons in Ireland (RCSI) University of Medicine and Health Sciences Dublin Ireland; ^7^ Department of Management & Marketing, Kemmy Business School University of Limerick Ireland; ^8^ Graduate School of Healthcare Management Royal College of Surgeons in Ireland Dublin Ireland

**Keywords:** Falls Management Exercise (FaME), falls prevention, mixed methods, older adults

## Abstract

**Background:**

Falls are the most common cause of hospitalisation amongst older adults in Ireland. Structured exercise programmes are recommended to manage falls, and the Falls Management Exercise (FaME) programme has been shown to reduce falls and improve physical function. However, FaME delivery within an Irish context needs to be explored to ensure it is implemented effectively and equitably.

**Objective:**

To explore key stakeholders' experiences and examine key outcomes during the early adoption of the FaME programme across three sites in Ireland.

**Design:**

A convergent mixed‐methods design was used to explore the early adoption of FaME across three sites in Ireland (Dublin, Kerry, and Sligo/Leitrim). Focus groups and interviews were conducted with key FaME stakeholders (clients, Postural Stability Instructors, service managers) to understand the experiences of local implementation. Baseline and outcome measures of falls and physical function contextualised the qualitative findings.

**Results:**

A census sample across the three study sites, 64 FaME Clients, 10 PSIs, and seven Service Managers were included. Themes described the Reach, Effectiveness, Adoption, Implementation, and Maintenance of FaME according to the RE‐AIM framework. All groups recorded improvements in physical function following FaME.

**Conclusions:**

This study highlights factors influencing the adoption of FaME within an Irish context and provides recommendations for future implementation. To ensure effective and sustainable delivery, commissioners should consider how FaME can be embedded within exercise and falls management pathways and establish partnerships with local community organisations to support service delivery.

**Patient or Public Contribution:**

Public representatives from *Age and Opportunity* (*n* = 16), a national organisation that advocates for valuable activities and services as we age, and clients of FaME (*n* = 4) were involved in the early protocol planning, scheduled meetings, co‐design phases, and analysis stages of the study. Their contribution influenced issues such as accessibility and acceptability of strength and balance programmes and options for dissemination. Representatives from *Age and Opportunity* reviewed the manuscript for readability.

## 1 Introduction

One in three people aged > 65 fall every year [1]. Fall‐related injuries are common, have negative effects on functional independence and quality of life, and are associated with increased morbidity, mortality and health‐related costs [1]. Recent Irish data shows that falls are the most frequent cause for hospital admission [2], with one in eight older people requiring medical attention for a fall [3]. Just 19% of fallers accessed physiotherapy [3], suggesting a missed opportunity to provide effective falls exercise as ‘treatment’ and as a ‘prevention strategy’ [4].

The Irish population aged over 65 is projected to rise to 1 million within 10 years and 1.6 million by 2051—further increasing the prevalence of falls [5]. Healthcare demands are already rising; Emergency Department attendances among people aged 75 and over have increased by 23.2% since 2023, with approximately half resulting in hospital admissions [6]. In 2023, 3983 people aged over 60 required hospitalisation with hip fractures resulting from low‐trauma falls [7]. The inpatient cost of hip fractures is estimated at €44 million, with an average inpatient stay of 17 days, accounting for 62,600 acute hospital days in 2021 [8]. The estimated cost of fall‐related injuries in Ireland, including primary, acute and social care, is projected to exceed €2 billion by 2030 [9].

The return on investment case for commissioning effective exercise for adults at risk of falling is clear, based on previous UK evaluations [1, 10]. Irish‐specific cost‐analysis is underway, and the data are not yet available. However, the growing burden of fall‐related healthcare costs in Ireland [9] suggests similar economic benefits could be realised.

FaME (Falls Management Exercise) is a multi‐component 24‐week group exercise programme, delivered by trained Postural Stability Instructors (PSIs). Each weekly session lasts approximately 60 min and includes (1): circulation boosting and mobility (2); static and dynamic balance training (reducing hand holds and base of support) (3); endurance training to improve stamina (4); strength training using body weight and resistance bands (5); preparation for and practice of getting down and up from the floor (backward chaining) and floor exercises (e.g., back extensions, side leg lifts); and (6) developmental stretches for flexibility and two to three Tai Chi moves. Intensity and challenge progress weekly. Participants are also prescribed a home‐based programme requiring approximately 1 h of additional exercises per week to support effective exercise dose [11, 12, 13, 14]. PSIs complete approximately 50 h of online and self‐directed learning plus 4 days of in‐person practical training. Further information on fidelity to FaME components and delivery is available [12, 13].

FaME has substantial patient benefits that not only include a reduction in falls, but also reduced fear of falling, improved physical function, balance confidence and quality of life, and an increase in habitual physical activity that approaches the national recommendations for physical activity and health [13, 14], ensuring benefits wider than falls prevention [15]. These benefits have been seen in randomised controlled trials with both sedentary, moderate risk, older adults (20%–30% fall each year) [14] and high‐risk older adults (3 or more falls in previous year) [11], and in UK‐based ‘real‐life’ implementation [13]. The current study aims to provide the first Irish ‘real‐life’ implementation data. In the United Kingdom, the National Institute for Clinical Excellence (NICE) have endorsed the UK's FaME Implementation Toolkit for use by commissioners and providers [16].

Based on the Health Service Executive's (HSE) 2008 National Strategy to prevent Falls and Fractures in Ireland's Ageing Population [17], the AFFINITY (Activating Falls and Fracture Prevention in Ireland Together) project was established in 2013 to coordinate a comprehensive, evidence‐informed approach to reducing harm from falls [18]. To improve the provision of evidence‐based exercise, AFFINITY supported the training of 103 PSIs (including both HSE Physiotherapists and private Exercise Professionals) to deliver FaME in Ireland. Our concurrent survey of the 103 trained PSIs shows that delivery of FaME is still emerging, with only 32 of the 67 respondents (48%) reporting that they were'actively delivering’ or 'intended to deliver’ FaME. Although much has been learned regarding the implementation of FaME in the United Kingdom [13, 19, 20], it is important to consider the implementation of FaME in an Irish context, where systems are different. The supporting structures to ensure FaME is effective and equitable need to be explored and optimised. Therefore, this study aims to describe and learn lessons from the early adoption of FaME within three sites across Ireland from the experiences of key stakeholders and key outcomes.

## 2 Methods

### 2.1 Study Design

A convergent mixed‐methods approach was used to explore the early adoption of FaME across three sites in Ireland: Dublin, Kerry and Sligo/Leitrim (Supporting Information S1: Appendix 1). This included gathering core data from the services, including uptake, reach, outcomes and maintenance of physical activity post‐FaME [21]. The data was gathered concurrently and integrated at the interpretation stage. The interview data not only provided the context for the early adoption of FaME but also gave a deeper insight into the quantitative outcomes. Qualitative data were analysed to understand how FaME was implemented locally within each site. The Consolidated Criteria for Reporting Qualitative Research (COREQ) and Standards for Reporting Qualitative Research (SPQR) guidelines have been followed for presentation of qualitative data [22, 23].

Ethical approval was granted by the Clinical Research Ethics Committee of the Cork Teaching Hospitals (CREC) [Ref Number EMC 4 (u) 14/05/2024 & ECM 3 (aaaa) 02/07/2024] and Sligo Research Ethics Committee [Ref 1019, 13/09/2024, 16/12/2024]. While the Dublin site temporarily lacked a local Research Ethics Committee at the time of the study, approval was formally granted by the HSE Research and Development Reform Team based on the ethical approvals obtained from the other participating sites' committees. Full ethical considerations were uniformly adhered to across all three sites, including obtaining informed written consent and ensuring identical participant rights, data protections and safety measures.

### 2.2 Participant Recruitment

Participants were recruited using census sampling at each study site, in which all eligible individuals within each stakeholder group were invited to participate. As this was a real‐world implementation study rather than an efficacy or effectiveness trial, sample size determination was not required. The sample sizes reflect the total number of stakeholders involved in FaME delivery and participation across the three sites during the study period.

### 2.3 Data Collection

Qualitative data were collected to assess the early adoption of FaME according to the reach of the service, effectiveness and adoption of the intervention implementation, sustainability of the service, and maintenance of physical activity post‐FaME. Data collection methods were tailored to the different stakeholder groups as outlined by the topic guides in Supporting Information S1: Appendix 2. All qualitative data collection was completed between September 2024 and May 2025 by members of the research team trained in qualitative research (R.M.C., K.T., C.E.). All interviews and focus groups lasted between 45 and 60 min. Topic guides remained consistent throughout the data collection period to ensure comparability across sites and participants.

In line with participatory action research principles, one PSI at each site acted as dual co‐researchers and participants, bringing valuable local knowledge to the study. To mitigate potential bias (1): FaME clients were interviewed by independent researchers not involved in service delivery (2); the research team practised reflexivity throughout data analysis, with regular debriefings to challenge preconceptions; and (3) two researchers independently analysed all transcripts.

#### 2.3.1 Interviews

The PSIs and service managers/partners were interviewed by RMC using semi‐structured topic guides. In most cases, there was no previous relationship with the interviewees, except with the site researchers. The interviews were held in‐person or online via a videoconference platform, depending on participant preference. All interviews were audio recorded and transcribed verbatim. Field notes were also collected during the interviews to provide context on the FaME sites and interviewee's role.

#### 2.3.2 Focus Groups

At each site, focus groups with three to nine FaME clients were conducted in‐person immediately following an exercise session in Week 12 (mid‐point of FaME) and Week 24 (end of the programme). These time points were selected to capture experiences during the programme's implementation and upon completion. For consistency in questioning, focus groups were conducted by the same members of the research team (K.T., C.E.); the researchers had no previous relationship with the FaME clients prior to the focus groups. All focus groups were audio recorded and transcribed verbatim.

#### 2.3.3 Quantitative Data

Finally, quantitative data were collected for all FaME clients at baseline and during the final FaME session to determine the effects of FaME. The following outcome measures were recorded by the PSIs (who were trained in conducting these measurements as part of their PSI training): Clinical Frailty Scale (CFS) [24]; EQ‐5D‐5L [25]; Falls Efficacy Scale‐International (FESI) [26]; Timed Up and Go (TUG) [27]; 30 s Sit‐to‐Stand (STS) [28]. The psychometric properties of these measures in older adult populations have been previously established [24, 25, 26, 27, 28] and are recommended by the World Falls Guidelines as core measures for falls prevention [1]. As this study did not aim to evaluate intervention effectiveness, assessors were not blinded to baseline results at follow‐up.

### 2.4 Data Analysis

#### 2.4.1 Qualitative Analysis

**Figure 1:**
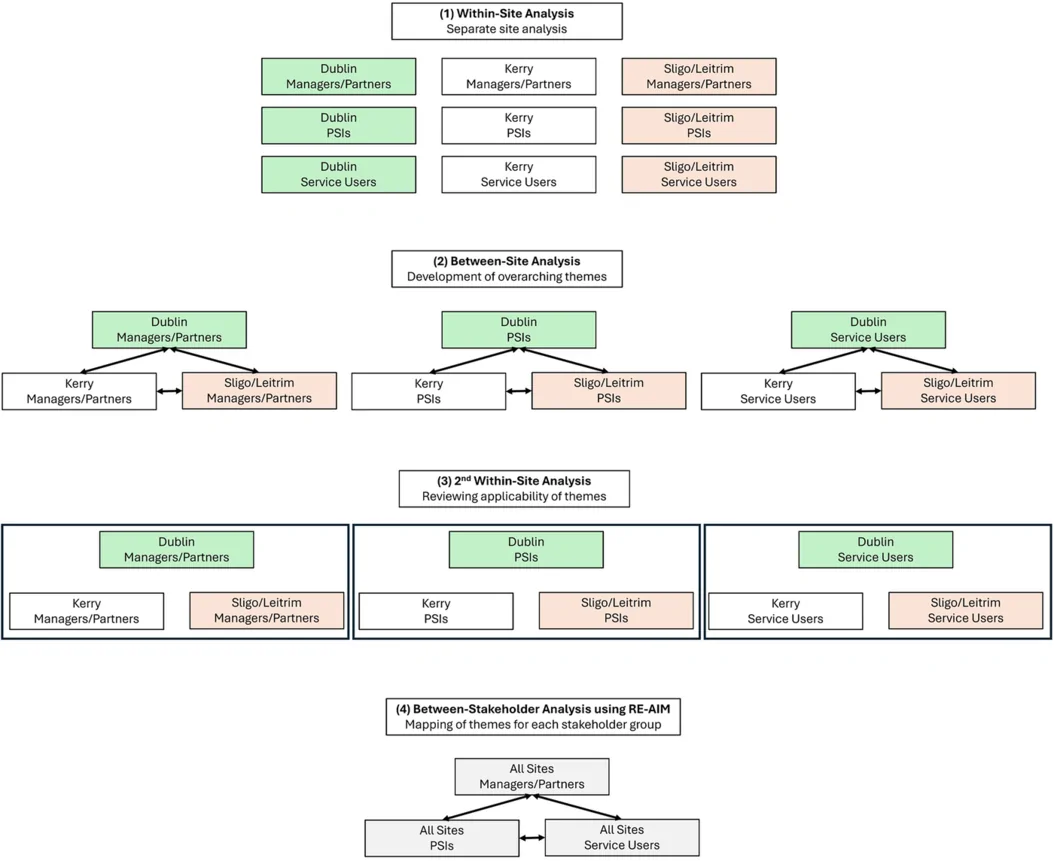
Process of multi‐site thematic analysis: The diagram illustrates the three‐phase process (1): within‐site analysis generating site‐specific themes (2); between‐site analysis identifying patterns across sites and developing overarching themes; and (3) stakeholder cross‐comparison and mapping to the RE‐AIM framework.

Interview and focus group data were analysed using the reflexive thematic analysis approach outlined by Braun and Clarke [29, 30]. Additionally, due to the multiple sites involved in this study, a framework for multi‐site qualitative analysis was followed to ensure relevant site‐specific context was retained within the thematic analysis process (Figure 1) [31].

For each interview/focus group transcript, two researchers (S.R., K.T., C.E., L.A., R.M.C.) independently read and inductively generated preliminary codes using NVivo 13 (QSR International). The coding pairs varied across transcripts, but one member of the research team (L.A.) coded all transcripts to ensure consistency. The second coder was typically one of the researchers who had conducted the focus group or interview and was familiar with the data. This approach combined consistency with familiarity with the context. Initial codes were compared and discussed between the paired coders; disagreements were resolved through discussion. The resulting codes were then presented to the wider research team for review, discussion, and refinement. This iterative process allowed multiple perspectives to be considered. Codes represented labels assigned to segments of data, whereas themes were interpretive concepts that linked multiple codes. Themes were developed through an iterative process of reviewing, combining, and refining codes across transcripts and sites. Transcripts were clustered for the coding process by research site and by stakeholder, meaning a unique set of codes was generated for each subset of data.

A within‐site analysis was then performed for each stakeholder group across the three research sites to generate an individual set of themes based on the agreed codes. A between‐site analysis was then performed by reviewing the commonalities and differences across the site‐specific themes for each stakeholder group. A pattern was defined as a theme that emerged in at least two of the three study sites. Where patterns of similar themes were identified between sites, overarching themes were then developed. Once the between‐site analysis was complete, the overarching themes were then reviewed by the wider research team to ensure that these themes still captured the meaning of the original data from each site.

Finally, the themes generated from the between‐site analysis were compared across groups and similar themes were combined where appropriate. These final themes were then mapped to the RE‐AIM framework [32] following the criteria outlined by Holtrop et al. [33].

#### 2.4.2 Quantitative Analysis

Baseline data were descriptively summarised using frequencies and medians with interquartile ranges due to the non‐normal distribution of data. Quantitative data were analysed descriptively to evaluate trends across the adopting sites. No inferential statistical testing was performed, as the study was not designed or powered to test hypotheses of efficacy. To assist readers in interpreting the clinical meaningfulness of the descriptive changes within this cohort, observed median differences were compared against published Minimally Important Differences (MIDs) for this population. These comparisons are intended solely as interpretive benchmarks and should not be interpreted as evidence of treatment efficacy or statistical significance.

## 3 Results

### 3.1 Demographics

Across the three study sites, 64 FaME clients (90% of FaME clients) participated in the FaME Ireland study. All 10 PSIs and all seven Service Managers/Partners participated in the study and were included in this analysis (Table 1). The median age of the FaME clients ranged from 77 to 86 years; most were women (72%) and had a CFS score of ≥ 4 (61%), indicating restrictions in usual activities. Clients in the Dublin site were older (median age 86 years) and more people had CFS scores of ≥ 4 (73%), indicating more restrictions in usual activities compared to Kerry (67%) and Sligo/Leitrim (50%). Differences in recruitment reflect site capacity. Drop‐out rates varied across sites, with Dublin having the lowest rate (14%), followed by Kerry (33%) and Sligo/Leitrim (36%). Simple review of baseline scores showed no clear differences in age, Clinical Frailty Scale scores, or functional measures (TUG, FES‐I) between completers and non‐completers. The reasons for the dropout variation were further explored in the qualitative findings, with programme gaps and venue changes identified as factors influencing adherence. PSIs included both physiotherapists (*n* = 4) and exercise professionals (*n* = 6) delivering FaME, while the service managers/partners worked in the HSE (*n* = 5) and community organisations (*n* = 2). Changes in outcomes following completion of the programme are described in Supporting Information S1: Appendix 3.

**Table 1:** Participant demographics.

FaME clients (*n* = 64)	Dublin (*n* = 15)	Kerry (*n* = 27)	Sligo/Leitrim (*n* = 22)
Age	86 [79.5–90.5]	79 [76–89]	77 [73.25–81]
Gender (M/F)	7/8	6/21	5/17
CFS			
*CFS 6*	0 (0%)	1 (4%)	0 (0%)
*CFS 5*	5 (33%)	7 (26%)	0 (0%)
*CFS 4*	6 (40%)	10 (37%)	10 (46%)
*CFS 3*	4 (27%)	6 (22%)	9 (41%)
*CFS 2*	0 (0%)	3 (11%)	2 (9%)
*CFS 1*	0 (0%)	0 (0%)	0 (0%)
*NR*	0 (0%)	0 (0%)	1 (4%)
Number of falls per participant (last 12 months)	1.9	1.5	2.0
EQ‐5D‐5L (Thermometer)	70 [45–80]	70 [60–80]	68 [50–70]
FESI	12 [10–15]	13 [10–15]	13 [11–18]
TUG (s)	12 [11–15]	13 [11–15]	13 [12–17]
30 s STS	8 [6–10]	7 [7–10]	10 [6–12]
**FaME instructors (*n*** = **10)**	**Dublin (*n*** = **5)**	**Kerry (*n*** = **3)**	**Sligo/Leitrim (*n*** = **2)**
Gender (M/F)	1/4	1/2	1/1
Role			
*Exercise professional*	3	2	1
*Physiotherapist*	2	1	1
**Service managers/partners (*n*** = **7)**	**Dublin (*n*** = **2)**	**Kerry (*n*** = **3)**	**Sligo/Leitrim (*n*** = **2)**
Gender (M/F)	1/1	2/1	0/2
Role			
*HSE Service Manager*	1	2	1
*HSE Falls coordinator*	0	0	1
*Community organisation manager*	1	1	0

*Note:* Values are presented as median [IQR] or as number (%) of participants. Number of falls per participant calculation: total number of falls in the group/total number of participants in the group. Abbreviations: CFS, Clinical Frailty Scale (ranges from 1 = *very fit* to 9 = *terminally ill*; no clients scored 7–9 in this sample); F, Female; FESI, Falls Efficacy Scale‐International; HSE, Health Service Executive; M, male; TUG, Timed Up and Go; 30 s STS, 30 s Sit‐to‐Stand.

### 3.2 Results by Theme

The 13 overarching themes from the stakeholder data were grouped according to the five domains of the RE‐AIM framework: ‘Reach’, ‘Effectiveness', ‘Adoption’, ‘Implementation’, and ‘Maintenance’. Themes were mapped to the RE‐AIM framework based on the perspectives of the stakeholder groups from which they emerged. Not all themes were represented across all stakeholder groups, reflecting the different roles and experiences of clients, PSIs, and service managers in the FaME programme. For example, service managers did not comment directly on adherence because they were not involved in day‐to‐day programme delivery. In contrast, clients did not comment on sustainability because they were not responsible for programme funding or management. This diversity of perspectives is a strength of the multi‐stakeholder design, providing complementary insights rather than competing claims.

The themes mapped to the RE‐AIM framework domains, and the stakeholder groups/study sites contributing to each theme are outlined in Table 2.

**Table 2:** Themes mapped to the RE‐AIM framework.

Stakeholder group	FaME site	Reach		Effectiveness		Adoption				Implementation			Maintenance	
		Referral in to FaME	Complexity of clients	FaME benefits to clients	Adherence to meet effective dose	Drivers for adopting FaME	Barriers to adopting FaME	Partnering with local organisations and agencies	Integration with physiotherapy services	Managing complexity	Accessibility and suitability of venues	Supporting PSIs	Keeping active after FaME	Sustainability
Service managers/partners	Dublin	✓				✓	✓	✓	✓	✓	✓	✓	✓	✓
	Kerry	✓				✓	✓	✓	✓		✓		✓	✓
	Sligo/Leitrim	✓				✓	✓	✓			✓	✓	✓	✓
PSIs	Dublin	✓	✓	✓	✓			✓	✓	✓	✓	✓	✓	✓
	Kerry	✓	✓	✓	✓			✓	✓	✓	✓	✓	✓	✓
	Sligo/Leitrim	✓	✓		✓			✓	✓	✓	✓	✓	✓	✓
FaME Clients	Dublin	✓	✓	✓	✓						✓		✓	
	Kerry	✓	✓	✓	✓						✓		✓	
	Sligo/Leitrim	✓	✓	✓	✓						✓		✓	

*Note:* Checkmarks (✓) indicate where a stakeholder group at each site contributed to the themes identified.

### 3.3 Reach

#### 3.3.1 Referral Into FaME (Table 3)

All stakeholder groups at each site discussed the referral process into FaME. At each site, referrals into FaME were predominantly made by HSE primary care physiotherapists according to all stakeholder groups interviewed. While the exact proportion of referrals from this source was not quantified, it was consistently identified as the primary referral pathway across all three sites. These primary care physiotherapists typically screened their waiting lists and patient caseloads to identify suitable clients for FaME. However, this process had the potential to miss suitable clients as many of the reasons for referral to physiotherapy were not falls‐related, despite a history and/or risk of falls. Therefore, their suitability for FaME was not initially clear from the referral. They were only identified as suitable when the physiotherapist opportunistically screened for falls risk.

Additionally, at some sites, due to inconsistent and uncertain funding for FaME, waiting lists were only generated at the point when new classes were scheduled to begin. While this approach minimised waiting times, it further limited the reach of the referral process. To mitigate these concerns, a rolling programme delivery was adopted by some sites to increase reach and avoid longer waiting times.

To further enhance the reach of referrals, alternative referral pathways were considered across sites (such as GP or self‐referrals). However, concerns around duplicate referrals for a single client and the ability to scale‐up FaME delivery to meet increased demand limited the wider early adoption of referral pathways beyond physiotherapy. Without opening alternative referral pathways, the local demand for FaME was unclear.

Therefore, *Reach* was limited during the early adoption stages by the existing referral process, with falls risk unclear from the referral details, inconsistent funding, and restricted eligible referrers to manage the potential demand/need for FaME.

#### 3.3.2 Complexity of Clients (Table 3)

The referral process into FaME required most clients to be already receiving healthcare, hence the FaME instructors highlighted that many clients were complex, living with frailty and multimorbidity—as confirmed by the participant demographics in Table 1.

Complexity was confirmed by the FaME clients—many of whom reported a history of frequent falls. Therefore, this led to FaME being primarily delivered as a secondary falls prevention intervention rather than for those with an intermediate falls risk, or as primary prevention (e.g., if they were avoiding activity through concern about falls).

Despite the increased frailty and clinical complexity, clients noted that while the high‐challenge components were demanding, such as backward chaining and floor exercises, they highly valued regaining their floor‐transfer skills.

Additionally, due to multimorbidity, clients highlighted that ongoing healthcare appointments and exacerbations of pre‐existing conditions often prevented full attendance at the sessions.

**Table 3:** Quotes supporting themes aligned with ‘Reach’ component of RE‐AIM.

RE‐AIM component	Theme	Illustrative quotes
Reach	Referral into FaME	‘Patients are referred sometimes specifically for falls…And then sometimes it's just a more global referral. But they are in the main, hitting our waiting list and from there [FaME referral] is happening ad hoc’. (Service Manager/Partner 7, Dublin) ‘We haven't really advertised yet. We're trying to get our own house in order before. There is a risk being inundated…we have to be able to cope with that’. (Servicer Deliverer 5, Dublin)
	Complexity of clients	‘We're working with a different cohort of people that are frail and that you have to be a little bit more aware of. And I suppose a lot of them coming in have a lot of underlying issues as well. So you to be aware of that too’. (PSI 2, Dublin) ‘[My referral] was the result of a lot of falls, a stroke and I had a serious accident years ago, and as a result my balance is very bad’. (FaME Client 2, Kerry) ‘I've stopped walking outdoors because I've had a lot of falls…I'm more confident walking at home than I am outside’. (FaME Client 26, Kerry) ‘I was terrified of going down on the ground…I have a knee replacement and I was uncomfortable trying to get up off the ground… [The PSIs] kept me doing it halfway and then do it a bit more. And then I was able to do it. I couldn't believe it. It wasn't so bad. I think it was the fear that was worse’. (FaME Client 53, Dublin)

### 3.4 Effectiveness

#### 3.4.1 FaME Benefits to Clients (Table 4)

Across all sites, clients consistently reported the benefits of FaME for confidence, mobility and falls. Clients reported less falls and improved balance and strength, which enabled them to return to activities of daily living that were previously impossible and led to improved confidence walking outdoors. It is important to note that these perspectives were gathered from clients who remained enroled in the programme; the views of those who discontinued may differ. Additionally, the focus group setting at FaME venues may have introduced social desirability bias, potentially leading to more positive responses. Clients also emphasised the importance and benefits of social interaction with others attending FaME. This was particularly impactful for clients who were previously socially isolated, as the group delivery of FaME provided them with social support.

#### 3.4.2 Adherence to Meet Effective Dose (Table 4)

Both FaME instructors and clients discussed adherence to the class and to home exercises. Factors impacting adherence either enabled or limited the clients' ability to meet the effective dose of exercise prescribed. For example, within sessions, clients reported finding the exercises challenged their strength and balance. Importantly, without the support of the PSIs, clients felt that they would be unable to perform a similar level of exercise independently.

The group delivery of FaME sessions also positively impacted adherence as clients reported feeling accountability to attend the class. However, in the absence of exercising with others, clients reported lower levels of motivation and adherence to their home exercise programmes.

To promote home exercise adherence, FaME instructors would encourage clients to integrate small bouts of exercises into their daily routine and usual activities, which clients felt helped with adherence.

Consistency in session delivery was also important to ensuring adherence. Across specific sites, gaps in programme delivery (e.g., due to venue availability or breaks over Christmas) and changing venues during the programme may have resulted in client drop‐out.

**Table 4:** Quotes supporting themes aligned with ‘Effectiveness’ component of RE‐AIM.

RE‐AIM component	Theme	Illustrative Quotes
Effectiveness	FaME benefits to clients	‘The class itself has transformed me and I'm only halfway through…I was in the garden hanging up the clothes. I always just stand on the cement but completely unconsciously, I walked on the grass. I was so excited, because it was major progress to walk on not solid ground’. (FaME Client 10, Kerry) ‘The week before last we were doing the floor exercise and that night I fell out of my bed… It was great knowing how to get up’. (FaME Client 45, Dublin) ‘You see my balance—it was very bad. And I was very stressed because of the falls, so I wasn't going out and I was afraid all the time… And now I'm not because the class has given me confidence and given me strength to hold my head up’. (FaME Client 39, Dublin) ‘I needed it. My husband died in October. I needed recovery…. I needed to get out of the house. It's a motivator’. (FaME Client 40, Dublin)
	Adherence to meet effective dose	‘There's a comfort in coming to a centre where, you know, there's experts and there… But I wouldn't do the level of exercise I do here at home. And I wouldn't have the same confidence of doing it at home’. (FaME Client 46, Dublin) ‘Home is challenging. I find, well… It's very difficult to motivate yourself’. (FaME Client 7, Kerry) ‘I might do a few exercises in the morning and then I might do one while you're waiting for the kettle to boil…I don't think of it as a chore, I kind of build it into my day’. (FaME Client 35, Sligo/Leitrim)

### 3.5 Adoption

#### 3.5.1 Drivers for Adopting FaME (Table 5)

Service managers and partners across all study sites discussed factors which positively influenced the adoption of FaME. They consistently highlighted the growing number of primary care physiotherapy referrals for older adults – the majority of which were presenting with frailty and risk of falls. (Service managers estimated that at two sites, 70% of clients attending primary care physiotherapy services were at intermediate or high risk of falls). FaME provided a suitable and evidence‐based falls management intervention for these clients and helped primary care services manage their waiting lists.

Aware of the unmet needs of falls prevention, service managers were strong advocates and important levers to adoption by raising awareness of the issue with local programme funders. Securing funding was critical to the adoption of FaME as the programmes were not permanently funded; instead, the managers had to apply annually for funding through HSE and local authority budgets. Funders having an interest in falls management helped facilitate the allocation of funding to FaME.

At one site, having a falls coordinator role was a key enabler as they helped service managers to raise awareness and support the application for funding by sharing their experience and knowledge around falls management. Furthermore, they are key to the seamless delivery of FaME, supporting the PSIs in the new adoption of FaME.

#### 3.5.2 Barriers to Adopting FaME (Table 5)

Service managers and external partners consistently identified constrained funding and limited resources as a primary barrier to implementing FaME across all sites. As FaME requires trained PSIs to deliver FaME, some service managers reported the challenges of identifying trained PSIs. They also experienced challenges in getting these PSIs released from their roles to deliver the programme—particularly HSE physiotherapists working in services with high waiting list demands and relatively low staffing levels to meet this demand.

The issue around identifying available staff was linked to the lack of a dedicated falls service within each site. For HSE physiotherapists, delivering FaME was not viewed as a core part of their role; therefore, they had to negotiate time within their usual clinical remit to deliver FaME.

The relative cost‐effectiveness of delivering FaME was critical to adoption, as when class attendances were low, there was a risk that physiotherapists would be required to stop delivering FaME.

#### 3.5.3 Partnering With Local Organisations and Agencies (Table 5)

Across all sites, the HSE partnered with local organisations and agencies to deliver FaME using different service delivery models (Supporting Information S1: Appendix 1). These organisations and agencies varied across sites and included local voluntary sector agencies, local councils and sports partnerships. These organisations were identified as understanding local community needs and would provide both PSI‐trained exercise professionals to deliver FaME, and source appropriate venues. Therefore, HSE service managers trusted these organisations in providing a quality service. At two sites, the HSE directly funded these local organisations to deliver FaME; at one site the HSE partnered with a local organisation to co‐fund FaME.

Most service managers acknowledged the value of FaME being HSE‐led (FaME was initiated to address the recognised gap in falls prevention programmes within the HSE, and service managers will have a greater influence in establishing the service they need). However, both they and the PSIs all highlighted that partnering with local organisations/agencies was critical to adopting FaME. Through providing trained exercise instructor PSIs, these local organisations/agencies ensured consistent programme delivery while minimising impact to HSE physiotherapy services by reducing the requirement for physiotherapy input. Without these partnerships, it was consistently acknowledged that the HSE would likely not be able to independently deliver FaME.

#### 3.5.4 Integration With Physiotherapy Services (Table 5)

Integrating FaME delivery within HSE physiotherapy services was viewed across all sites as a factor which aided programme adoption. From the perspective of service managers, FaME was seen to provide a consistent option for older adults with a risk of falls. This complemented existing services by providing an evidence‐based programme to which physiotherapists could refer. Ultimately, this positively impacted waiting lists by reducing the number of physiotherapy appointments provided to older adults.

The physiotherapist PSIs also recognised this benefit; however, this was viewed in the context of balancing competing demands. Whilst they acknowledged FaME provided an effective service, their involvement in the delivery of FaME limited their usual clinical roles. The physiotherapists were aware that if primary care waiting lists were to increase, management would no longer support their involvement in FaME delivery.

As part of integrating FaME delivery within physiotherapy services, interviewees recognised that referral options were needed for those currently unable to do FaME—such as Otago [34]—and to other established exercise programmes for clients after FaME as part of a falls management pathway. This pathway currently does not exist across sites.

**Table 5:** Quotes supporting themes aligned with ‘Adoption’ component of RE‐AIM.

RE‐AIM component	Theme	Illustrative quotes
Adoption	Drivers for adopting FaME	‘This was a high‐need area, with a lot of risk associated by not addressing it. So, we weighed up how we best use our resources and decided to prioritise into this area’. (Service Manager/Partner 7, Dublin) ‘Our head of service sees it as a very important prevention piece, so we're very fortunate in that. And it's been funded, really from savings…it's not core Health and Wellbeing funding’. (Service Manager/Partner 2, Sligo/Leitrim) ‘[The falls coordinator] makes a huge difference. Even though it's fragmented, she pulls it all together. It wouldn't happen without her’. (PSI 10, Sligo/Leitrim)
	Barrier to adopting FaME	‘We have another physio who is a trained PSI but who hasn't delivered a programme due to the demand of the work in the area’. (Service Manager/Partner 1, Sligo/Leitrim) ‘It absolutely needs to be tackled. But it doesn't have a home… Initially it felt like I'm abandoning my day‐to‐day management of my waiting list in lieu of delivering FaME’. (Service Manager/Partner 7, Dublin) ‘It is important for us to show that value for money piece in terms of at least keeping the numbers as high as you can’. (Service Manager/Partner 6, Dublin)
	Partnering with local organisations and agencies	‘The advantage is you're working with an organisation that have their feet on the ground and know the needs, they can respond much better and can be more flexible’. (Service Manager/Partner 3, Kerry) ‘We've been very fortunate to partner with these excellent organizations…to develop models of practice that we could not do on the HSE ourselves’. (Service Manager/Partner 3, Kerry) ‘But we're very fortunate that over the years we have a very good working relationship with the sports partnerships. It really is a key enabler for the program in the area’. (Service Manager/Partner 2, Sligo/Leitrim) ‘I think we're getting really good at building connections with community partners, and I think our community partners are getting really good at seeing where there's a need and and addressing that need’. (Service Manager/Partner 6, Dublin)
	Integration with physiotherapy services	‘It's certainly an advantage to us. A typical patient…. we would probably see them maybe 3‐4 times over a two‐month period to try to progress a rehab program…But if we can refer them on to FaME then that's a huge benefit’. (Service Manager/Partner 5, Kerry) ‘At the moment, there's only positive feelings toward FaME from within the physio service because of the way it's set up. If it's seen in any way to potentially produce additional referrals then that kind of sentiment towards it changes’. (Service Manager/Partner 5, Kerry)

### 3.6 Implementation

#### 3.6.1 Managing Complexity (Table 6)

As previously highlighted, as most referrals into FaME were from primary care physiotherapists, many FaME clients had complex clinical presentations. Therefore, FaME instructors consistently highlighted the importance of adapting exercise delivery to suit the needs of the clients. This ensured safety while optimising exercise dose. However, FaME instructors found this challenging when sessions were comprised of clients with mixed levels of ability in the one class, despite this being covered in their original training. Some PSIs managed to implement two levels of classes (higher and lower functioning) within services. To support service delivery, FaME sessions were delivered by two PSIs in two study sites. This was viewed as critical by the PSIs due to the challenge of managing complex clients within sessions—particularly with challenging exercises such as floorwork. At these two study sites, PSIs did not feel confident delivering sessions alone unless the class sizes were reduced or the clients were less complex. The third site operated with a single PSI per session while supported by a Falls Coordinator. The client experiences and outcomes at this site were consistent with the other sites, suggesting that while two PSIs may be preferred, single‐PSI delivery can be viable with appropriate supports and risk management.

In recognising the complexity of clients, exercise professionals also highlighted the value of physiotherapy support. Across sites, the nature of support varied from physiotherapists leading the assessment of clients (due to exercise professionals being unsure about complex medical histories) and physiotherapists delivering FaME sessions alongside the exercise professionals. Additionally, across all sites, exercise professionals highlighted the value of linking with physiotherapy services—either to redirect clients unsuitable for FaME, or to receive advice regarding changes in client presentation during FaME.

#### 3.6.2 Accessibility and Suitability of Venues (Table 6)

Another key consideration in the early adoption of FaME was the selection of appropriate venues. All stakeholder groups identified the need for venues to be accessible, suitable for exercise class delivery (in terms of size, lighting, acoustics), and served by transport links. It was also important that the same venues were used across the full programme to promote adherence. Where possible, clients also highlighted the importance of scheduling sessions during daylight hours, while avoiding peak travel times.

#### 3.6.3 Supporting PSIs (Table 6)

FaME Instructors highlighted the importance of support when implementing the programme. This was particularly relevant for those with less experience in FaME delivery, who often felt less confident. A key form of support identified across all sites was establishing a Community of Practice where FaME deliverers could support and learn from each other.

FaME instructors also required support around class preparation. The time required to set up the venues pre‐session and complete paperwork post‐session had to be considered within the time allocated for FaME delivery. Simplification of paperwork and the provision of handovers between sessions helped with class preparation.

**Table 6:** Quotes supporting themes aligned with ‘Implementation’ component of RE‐AIM.

RE‐AIM component	Theme	Illustrative quotes
Implementation	Managing complexity	‘If we have someone who is very high level and someone at a lower level, we may have to slow things down or not do much for the lower level…so different levels can be hard to manage’. (PSI 9, Sligo/Leitrim) ‘They are a very vulnerable cohort, so all it takes is one person not feeling great…so that's often a challenge for us…the risks are high with this group, but we do need to progressively challenge them. So, it is nice to have a second person there’. (PSI 5, Dublin) ‘You assess the client…but then things pop‐up for the client two weeks later…Throughout the program, things can change dramatically. So if there's just an exercise facilitator, they need to have direct access to a physio to ask ‘what should I do?’ (PSI 8, Kerry)
	Accessibility and suitability of venues	‘Last week I thought was terrible. It was totally black…you don't realise an hour leaves it so dark outside. And it even crossed my mind, should I change the 2:00 class?’ (FaME Client 35, Sligo/Leitrim)
	Supporting PSIs	‘I suppose you're working with a different cohort of people that are fragile and that you have to be a little bit more aware of…So at the start that can be quite daunting’. (PSI 8, Kerry)

### 3.7 Maintenance

#### 3.7.1 Keeping Active After FaME (Table 7)

Maintenance was first considered in relation to clients maintaining the gains of FaME upon completion. All stakeholder groups discussed the availability of local exercise opportunities to keep active after FaME. The availability varied between sites, with more opportunities available in urban settings. However, generally, clients felt there were limited opportunities to maintain or progress exercise after FaME. Most available exercise programmes were either a regression on FaME (e.g., seated exercise) or were too advanced. Participants across different sites shared this experience and highlighted a broader issue: the lack of suitable existing exercise opportunities for older adults with a history of/or at risk of falls. This suggests systemic barriers to post‐FaME exercise participation rather than isolated incidents. Despite the limited available opportunities, clients consistently reported motivation to continue exercising post‐FaME—preferably within a similar group setting.

Typically, clients found appropriate exercise options informally through friends and family. In the absence of other exercise opportunities, many clients expressed a preference to start FaME again—with some having already completed multiple deliveries of the programme. Many clients expressed that they were willing to pay a weekly nominal fee to continue attending FaME, and where exercise instructor PSIs were involved, there was the potential for them to offer these sessions on a ‘pay as you go’ basis.

#### 3.7.2 Sustainability (Table 7)

Maintenance was also discussed by service managers and FaME instructors in the context of programme sustainability. For FaME to be sustainable, service managers highlighted the need for the programme to be effective—both in terms of falls prevention and the impact on existing physiotherapy services. Being able to demonstrate this impact was critical, as higher‐level HSE management support was considered essential to sustaining FaME.

The lack of a dedicated local falls strategy negatively impacted the sustainability of FaME; without clear ownership and leadership of falls management, its long‐term sustainability was dependent on successful annual funding applications. If this funding was not received, then the programme was halted. This highlighted the critical role of the falls co‐ordinator within one of the study sites, as their advocacy and awareness raising around falls management helped promote the sustainability of FaME.

Finally, retaining PSIs was critical to ensuring sustainability. Models of delivery led by exercise professionals rather than physiotherapists were viewed as more cost‐effective. However, as exercise professionals were employed on ‘zero‐hour’ contracts across two of the study sites, this presented a challenge to retaining PSIs long‐term.

**Table 7:** Quotes supporting themes aligned with ‘Maintenance’ component of RE‐AIM.

RE‐AIM component	Theme	Illustrative quotes
Maintenance	Keeping active after FaME	‘Because I had fallen someone had told me about the classes down at the Community Centre. I went down there on my own…and they said no can't take you ‐ too much of a liability because I was using a walking stick’. (FaME Client 31, Kerry) ‘I'll go back to check if there is an alternative ‐ something similar to this here…But I would prefer to stay here if I could’. (FaME Client 53, Dublin)
	Sustainability	‘We've been lucky that the network manager has allowed us to decide how to use the resources and stick to this goal of delivering [FaME]. And it hasn't resulted in longer waiting lists so far, so good’. (Service Manager/Partner 7, Dublin) ‘I think there's way more advocacy needed around that whole area…We need more funding coming in…Ideally, we should be running more of these, but at the same time, the funding has to come from somewhere’. (Service Manager/Partner 6, Dublin) ‘I'm being released from primary care to do this. I suppose it isn't necessarily part of my role to run the falls classes. So I had to get permission from my manager to be part of this programme because I'm not under a falls team’. (PSI 9, Sligo/Leitrim)

## 4 Discussion

This mixed‐methods study explored the early adoption of FaME within three sites across Ireland. These sites represented three distinct regions, and the perspectives of service managers, PSIs, and FaME clients were considered across each programme. As the first study to explore the adoption of FaME in Ireland, these findings highlight key considerations for the local implementation of FaME and can inform future service delivery.

The current HSE Falls Prevention Framework recommends that people at risk of falls should undertake a multicomponent exercise programme—such as FaME—to reduce falls risk [35]. This recommendation aligns with the HSE Physical Activity Pathways, in which prescribed community‐based physical activity programmes should be available to manage chronic health issues such as falls [15]. However, despite the presence of a falls coordinator at one site, none of the three study sites had a dedicated falls service. The falls coordinator at Sligo/Leitrim worked across multiple services, managing adults at risk of falling, but did not operate within a specific falls service. This lack of dedicated falls services was a key challenge to adopting FaME. Similar challenges within a UK context have been reported [19, 20]. Without a dedicated falls service, ownership and responsibility for delivering FaME is unclear, and early adoption was ad‐hoc, relying upon local health professionals to advocate for FaME to address the unmet need for falls prevention services. This also challenged the sustainability of FaME, as without dedicated staff assigned to a falls service, those involved in delivering FaME had to manage the competing demands of their primary clinical responsibilities. Moving forward, it is recommended that evidence‐based falls prevention interventions such as FaME should be integrated within a national pathway to ensure a sustainable service delivery that meets the growing demand for falls management in Ireland [3].

Alongside previous research [19], this study also found that the availability of resources was critical to the adoption of FaME. In particular, the sustainability of funding was highlighted as a key issue since the FaME programmes in Ireland were not permanently funded and instead were dependent on the approval of annual funding applications. Consequently, most of the exercise professional PSIs delivering FaME were employed on zero‐hour contracts. This limited the time available to deliver FaME, with essential components such as post‐class social time often omitted despite it importantly addressing social isolation [36, 37]. Therefore, it is recommended that when organisations consider sustainable funding models for FaME, they determine the appropriate staffing and time resources required to deliver FaME with fidelity. Based on the findings from this study, this should include consideration of (1): adequate time for class preparation and administration beyond the session itself (2); support for two PSIs per session for complex client groups, or ways to address the complexity of the client groups; and (3) opportunities for Communities of Practice to support PSI confidence and skill development. Further research is needed to establish precise resource requirements and economic models. Additionally, further work should explore sustainable staffing models for PSIs, as the delivery of programmes is dependent upon the retention of trained staff [20]. This includes exploring partnerships with local organisations that employ exercise professional PSIs, such as those used across the included study sites.

To support the implementation of FaME, key learning from this study emphasised the importance of integrating FaME with existing healthcare services—which in the context of this study was HSE primary care physiotherapy services. Through providing an evidence‐based falls management intervention for those at risk of falls, FaME complemented existing physiotherapy services and ultimately had a positive impact on waiting lists by reducing the number of physiotherapy appointments required by older adults. However, the reach of referral pathways should be considered to maximise access to FaME programmes. Over‐reliance on a single source of referrals was limiting access and creating referral bottlenecks. Additionally, due to the nature of physiotherapy referrals, FaME programmes in Ireland were primarily being delivered as a secondary falls prevention intervention for those at high risk of falls rather than directly targeting those with intermediate falls risks. Therefore, in line with the World Falls Guidelines, organisations should consider multiple sources of referral (e.g., from a range of healthcare professionals alongside self‐referral) to maximise reach and identify both those at high and intermediate falls risks [1]. Additionally, a single central point of referral should be developed with clear signposting to improve healthcare professional and public awareness.

While the clients included in this study reported improvements in their mobility and balance, they faced challenges in maintaining these gains upon programme completion. Although most clients expressed the motivation to continue exercising post‐FaME, the lack of suitable exercise options restricted their ability to do so. This pattern of reduced physical activity is often observed following exercise programmes for older adults [38, 39, 40]. While behaviour change techniques can support older adults to maintain physical activity levels [41], interventions are also required to address structural barriers to physical activity, such as the lack of suitable exercise opportunities. For example, PSIs can offer private maintenance programmes to help motivate and support older adults to remain physically active after FaME [42]. Such programmes need to be embedded within the HSE Physical Activity Pathways and provide a clear route for people to progress into and out of FaME [15].

## 5 Study Limitations

Although the three sites included in this study have diverse regional characteristics, it is unclear whether these findings represent the wider experiences of adopting FaME across other regions of Ireland. The three sites were selected purposively rather than randomly, limiting generalisability to other Irish regions or falls prevention programmes. The sample sizes across sites were small (*n* = 15, 27, 22) and reflect the real‐world operational boundaries of early adoption. Site differences in age, frailty, and drop‐out rates may influence the generalisability of findings. While baseline differences were not adjusted for statistically, the qualitative data provide context for understanding these variations. Multi‐site thematic analysis was used to identify consistent themes across sites and support the transferability of findings, there may however, be additional perspectives not captured by any of the locations included in this study. Additionally, due to the specific nature of FaME, these findings may have limited transferability to other falls prevention interventions.

The small number of PSIs (*n* = 10) and service managers (*n* = 7) means that individual perspectives from these stakeholder groups carry more weight in the qualitative findings. However, this is expected given the group‐exercise nature of FaME, where the number of clients will naturally exceed the number of service providers. The multi‐site design and inclusion of multiple stakeholder perspectives help mitigate the influence of any single individual's view.

The client interviews also only included those attending FaME and may not represent the experiences of those who discontinued the programme. The focus group setting at FaME venues may have introduced social desirability bias, leading participants to provide more positive responses. The falls history was self‐reported over 12 months, which is subject to recall bias. Furthermore, with a lack of long‐term follow‐up, it is unclear how experiences and outcomes change post‐programme.

Due to the small sample sizes, the quantitative data should be treated as strictly descriptive. The application of MIDs in this study is intended solely as an interpretive benchmark for clinical relevance within this specific group, rather than as evidence of statistical power or generalisable treatment efficacy.

Finally, in line with participatory action research principles, the study was strengthened by the site‐researchers acting as dual co‐researchers and participants, ensuring the evaluation remained focused on adopting FaME in Ireland. Independent cross‐coding during thematic analysis and team reflexivity was employed to counterbalance potential bias.

## 6 Conclusions

This study highlights factors influencing the adoption of FaME within an Irish context and provides recommendations to those responsible for reducing the rate and risk of falls in Ireland. Considering the national importance of falls management, health service providers should consider how FaME can be embedded within exercise and falls management pathways. Additionally, partnerships with community organisations should be explored to support sustainable service delivery. Finally, sustainable provision of resources should be considered to ensure FaME is delivered consistently and with fidelity. Future research should evaluate the effectiveness and efficiency of service delivery models to support national adoption.

## Author Contributions

**Scott Rooney:** formal analysis, methodology, writing (original draft preparation), writing (review and editing). **Leanne Ahern:** data curation, formal analysis, investigation, methodology, writing (review and editing). **Edel Brennan:** conceptualisation, data curation, funding acquisition, investigation, methodology, resources, writing (review and editing). **Eibhlis Cahalane:** conceptualisation, data curation, funding acquisition, investigation, methodology, resources, writing (review and editing). **Vanda Cummins:** conceptualisation, data curation, funding acquisition, investigation, methodology, resources, writing (review and editing). **Caroline Eldridge:** data curation, formal analysis, investigation, methodology, writing (review and editing). **Frances Horgan:** conceptualisation, funding acquisition, methodology, project administration, supervision, writing (review and editing). **Dawn A. Skelton:** conceptualisation, formal analysis, funding acquisition, investigation, methodology, supervision, writing (original draft preparation), writing (review and editing). **Éidín Ní Shé:** data curation, formal analysis, investigation, methodology, writing (review and editing). **Katherine Thackeray:** data curation, formal analysis, investigation, methodology, writing (review and editing). **Boglarka Vig:** conceptualisation, data curation, investigation, methodology, resources, writing (review and editing). **Ruth McCullagh:** conceptualisation, data curation, formal analysis, funding acquisition, investigation, methodology, project administration, supervision, writing (original draft preparation), writing (review and editing).

## Conflicts of Interest

Dawn A. Skelton is a Director of Later Life Training, a not‐for‐profit organisation that provides training in the delivery of FaME and Otago to health and fitness professionals in the UK and Ireland.

## Supporting information


Supporting File

## Data Availability

The data that support the findings of this study are available on request from the corresponding author. The data are not publicly available due to privacy or ethical restrictions.
