# Supplementary material for: Lessons Learned in the Early Adoption of FaME Across Ireland: A Convergent Mixed‐Methods Implementation Study (FaME Ireland)
Source: Health Expect. 2026 Sep 7;29(5):e70860. doi: 10.1111/hex.70860 (PMC13548410; doi:10.1111/hex.70860)
Supplement: Supplementary file 1 [file HEX-29-e70860-s001.docx]

**Appendix 1 Overview of study sites**

|  | **Dublin** | **Kerry** | **Sligo/Leitrim** |
| --- | --- | --- | --- |
| **Programme duration (weeks)** | 24 | 24 | 24 |
| **Weekly sessions** | 1x | 1x | 1x |
| **Number of PSIs (per session)** | 2 | 2 | 1 |
| **Details of PSIs** | Physiotherapist (first 6 weeks); Exercise professionals (final 18 weeks) | Physiotherapists and exercise professionals (full 24 weeks) | Physiotherapist (first 6 weeks); Exercise professionals (final 18 weeks) |
| **Funders** | HSE and local council | HSE | HSE |
| **Local Partnering Organisation** | Dublin City Council | Local voluntary agency | Local Sports Partnership |
| **Geographical location** | Urban | Rural | Rural |
| **FaME locations per site** | 2 | 2 | 1 |
| **Venue** | Community centre | Community centre | HSE (first 6 weeks); Community centre (final 18 weeks) |

**Appendix 2 Interview/focus group topic guides**

1. ***Topic Guide for focus groups with FaME Clients***

| Welcome and thank participants for attending and participating in the interview.  Explain the aim of the interview is to explore the strengths, weaknesses, opportunities and threats they perceive in the early stages of delivering the program.  Establish the ground rules [confidentiality, respect and speaking over someone else). Explain that we would appreciate respectful, open, and honest discourse in order to identify what is working well and what needs to be changed and identify realistic and reasonable solutions. | |
| --- | --- |
| General introduction | 1. Ask them to introduce themselves and tell us how they heard of the programme. [Note names to encourage involvement, or to identify who is saying what]. 2. In general, how are things going? Are you enjoying the program? |
| Reach & Equity | 1. Which health professional told you about the programme? Did you have to wait long to get into the classes? 2. Were there barriers (transport. scheduling, health) that affects your ability to attend the programme? 3. What makes it possible for you to attend (location, support from others, transport etc.) |
| Treatment fidelity barriers and opportunities | 1. How confident are you all doing the exercises in the class? 2. Are there any exercises that are particularly challenging or intimidating? 3. What about the floor work, or getting down to the floor? 4. What about doing the exercises at home? 5. Have you noticed any changes in your ability or confidence since you have started the classes? 6. What impact, if any, has the class had on your overall lifestyle or health? |
| Sustainability | 1. Do you think the programme is worth keeping? Why? 2. How could we make these classes more available? |
| Egress | 1. At the end of 26 weeks, you will finish the program. We endeavor to encourage you to maintain physical activity and independence after the program. 2. Do you know of any local services or exercise groups that may be suitable? 3. Do you have any experience, or have you contacts for these groups? 4. How do you feel about going on to a different exercise program? What would concern you about it? |
| Conclusion | 1. Is there anything else you would like to discuss are you feel that we may have left out? |
| Thank the participants for the interview and the opportunity to gain insight into their experience. Summarize your interpretation of the interview for member checking. Invite them to add any additional information over the next week if required. If SSI, remind them that they can withdraw any information for two weeks after this interview. | |

2. ***Topic Guide for interviews with PSIs***

| Start recording. State your name, date, time, site, and interviewees name.  Thank participants for attending and participating in the interview.  Explain the aim of the interview is to explore the strengths, weaknesses, opportunities and threats they perceive in the early stages of delivering the program.  Explain that we would appreciate respectful, open, and honest discourse in order to identify what is working well and what needs to be changed and identify realistic and reasonable solutions. | |
| --- | --- |
| General introduction | 1. Which professional group [physio/ exercise instructor] 2. What organization do you work for? 3. In general, how are things going? Are you enjoying the program? |
| Treatment fidelity barriers and opportunities | 1. Firstly, can you walk us through the process please? When a participant comes into the room... Elaborate the room setting, equipment, exercises, finish up, estimate of the PSI’s overall time spent per class. 2. How many participants are in your class? How are you finding delivering the program with this number of participants? [Discuss safety, achievement fidelity, and confidence in delivering the program?] 3. How do you guide and support the home exercises? 4. How do you find the backward chaining and floorwork? 5. Can you think of simple solutions to provide sufficient support to PSIs? 6. Can you discuss the barriers, or frustrations you have experienced on this occasion to treatment fidelity. This may include people resources and equipment resources. |
| Sustainability | 1. What concerns have you about sustaining the program going forward. This may include people of resources, working relationship between physiotherapists and PSI's, and equipment resources. 2. In your current set-up, is it viable for you to continue to deliver FaME? [probe what makes it viable/not viable, mode of employment etc]. 3. You work closely with the fellow PSIs here. Why does it work well? What are the advantages of the partnership? Can you think of any disadvantages? Any solutions? 4. If there were no limits to what was needed, in other words blue sky thinking, what do you think would help run the program in the best way possible. |
| Reach & Equity | 1. What have the referral rates been like? 2. Have the participants been appropriate for the classes? 3. At the moment, all the referrals come through physio. If this was more widely accessible, how do you think it could be opened up? Do you think there's a demand/need for this service. 4. Are you managing to reach everybody who could benefit from the class? Is there a group of people that we are “missing”? How best could we reach them? |
| Egress | 1. At the end of 26 weeks, participants will finish the program. We endeavor to encourage them to maintain physical activity and independence after the program. 2. How do you encourage them to keep up with the exercise after the programme? 3. Are there any local services or exercise groups that may be suitable for the participants? 4. Do you think the participants would be interested? Have they discussed options? 5. Do you have any experience, or have you contacts for these groups? |
| Conclusion | 1. Is there anything else you would like to discuss are you feel that we may have left out? |
| Thank the participant for the interview and the opportunity to gain insight into their experience. Summarize your interpretation of the interview for member checking. Invite them to add any additional information over the next week if required. Remind them that they can withdraw any information for two weeks after this interview. | |

3. ***Topic Guide for interviews with Service Managers/Partners***

| Thank participants for attending and participating in the interview.  Explain the aim of the interview is to explore their knowledge and awareness of Falls Prevention Services in their local area.  Explain that we would appreciate respectful, open, and honest discourse in order to identify what is working well and what needs to be changed and identify realistic and reasonable solutions. | |
| --- | --- |
| General introduction | 1. Can you tell me what is your professional role, and where do you work? 2. What is your role in caring for older people? 3. What organization do you work for? 4. In general, do you work with many people who are at risk of falling? |
| Their awareness of falls prevention services in the area | 1. Can you tell me about falls prevention services in the area? [expand points of interest and look at overlap] 2. Do you care for people who may benefit from falls prevention classes? [develop an understanding of the type of person] How do you access falls prevention for them [referral routes, access, etc?] 3. Do you have any awareness of how long it takes to access them [class provision, access, availability, waiting lists?] 4. Do you have any awareness of their satisfaction of the services? 5. What do you think of the services? 6. Do you have any suggestions as to how services could be improved? |
| Exercise classes | 1. What general exercise classes, or ways for older people to be physically active available in the area? 2. Do you refer to these services [explore their motivation and how accessible they are]. |
| Reach & Equity | 1. Do you think there's a demand/need for this service? 2. Are there other options available for fallers? 3. Can you tell us about people who would benefit from falls prevention classes but are not suitable [explore the type of person who is unable to join classes]. What else is available for them? What are the bottlenecks in the service provision? 4. Is there a group of people that are “missing out”? |
| Conclusion | 1. Is there anything else you would like to discuss are you feel that we may have left out? |
| Thank the participant for the interview and the opportunity to gain insight into their experience. Summarize your interpretation of the interview for member checking. Invite them to add any additional information over the next week if required. Remind them that they can withdraw any information for two weeks after this interview. | |

**Appendix 3 Changes in client outcomes following FaME**

|  | **Dublin (n=15)** | **Kerry (n=27)** | **Sligo/Leitrim (n=22)** |
| --- | --- | --- | --- |
| Drop-outs, n (%) | 2 (14%) | 8 (33%) | 8 (36%) |
| Number of falls per participant (last 6 months) | 1.0 | 0.2 | 0.2 |
| EQ-5D-5L (Thermometer) | 10 [10-17] **↑*** | 0 [0-7.5] **↔** | 5 [0-23] **↑** |
| FESI | -1 [-2.5- -1] **↑** | -5 [-10- -5] **↑** | -3 [-6- -3] **↑** |
| TUG (s) | 1 [-0.5-1.4] **↓** | -3 [-3.5- -1] **↑*** | -2.8 [-4.5- -2.4] **↑*** |
| 30s STS | 1 [2-3] **↑** | 3 [-2-3] **↑*** | 2 [0-4] **↑*** |

Values are presented as median difference [IQR] relative to baseline or as number (%) of participants

Number of falls per participant calculation: total number of falls in the group / total number of participants in the group

↑ represents an improvement in outcome measure score; ↓ represents a worsening in outcome measure score; ↔ represents no change

* Median difference exceeds the minimal clinically important difference for the: EQ-5D-5L = 6.5 (Cheng et al. 2024); TUG = 1.6 (Low et al. 2022); 30s STS = 2 (Wright et al. 2011): These comparisons are intended solely as interpretive benchmarks and should not be interpreted as evidence of treatment efficacy or statistical significance

FESI, Falls Efficacy Scale-International; TUG, Timed Up and Go; 30s STS, 30s Sit-to-stand

References

L. J. Cheng, L. A. Chen, J. Y. Cheng, M. Herdman, and N. Luo. “Systematic Review Reveals that EQ-5D Minimally Important Differences Vary With Treatment Type and may Decrease with Increasing Baseline Score.” *Journal of Clinical Epidemiology* 174 (2024): 111487. https://doi.org/10.1016/j.jclinepi.2024.111487.

D. C. Low, and G. S. Walsh. “The Minimal Important Change for Measures of Balance and Postural Control in Older Adults: A Systematic Review.” *Age Ageing* 51, no. 12 (2022): afac284. https://doi.org/10.1093/ageing/afac284.

A. A. Wright, C. E. Cook, G. D. Baxter, J. D. Dockerty, and J. H. Abbott. “A Comparison of 3 Methodological Approaches to Defining Major Clinically Important Improvement of 4 Performance Measures in Patients With hip Osteoarthritis.” *Journal of Orthopaedic Sports Physical therapy* 41, no. 5 (2011): 319-327. https://doi.org/10.2519/jospt.2011.3515.
